# Mining, analyzing, and integrating viral signals from metagenomic data

**DOI:** 10.1186/s40168-019-0657-y

**Published:** 2019-03-19

**Authors:** Tingting Zheng, Jun Li, Yueqiong Ni, Kang Kang, Maria-Anna Misiakou, Lejla Imamovic, Billy K. C. Chow, Anne A. Rode, Peter Bytzer, Morten Sommer, Gianni Panagiotou

**Affiliations:** 1 Systems Biology & Bioinformatics Group, School of Biological Sciences, Faculty of Sciences, The University of Hong Kong, Hong Kong, Hong Kong, Special Administrative Region of China; 2 Department of Infectious Diseases and Public Health, The Jockey Club College of Veterinary Medicine and Life Sciences, City University of Hong Kong, Hong Kong, Hong Kong, Special Administrative Region of China; 3 School of Data Science, City University of Hong Kong, Hong Kong, Hong Kong, Special Administrative Region of China; 4 Department of Systems Biology and Bioinformatics, Leibniz Institute for Natural Product Research and Infection Biology, Hans Knöll Institute (HKI), Beutenbergstraße 11a, 07745 Jena, Germany; 5 Bacterial Synthetic Biology Section, Novo Nordisk Foundation Center for Biosustainability, Technical University of Denmark, Kemitorvet, 2800 Kongens Lyngby, Denmark; 6 School of Biological Sciences, Faculty of Science, The University of Hong Kong, Hong Kong, Hong Kong, Special Administrative Region of China; 7 Department of Medicine, Zealand University Hospital, Køge, Denmark; 8 Department of Clinical Medicine, University of Copenhagen, Copenhagen, Denmark; 9 Department of Microbiology, Li Ka Shing Faculty of Medicine, The University of Hong Kong, Hong Kong, Hong Kong, Special Administrative Region of China

**Keywords:** Phage, Metagenome, Phage-host interaction, Antibiotics

## Abstract

**Background:**

Viruses are important components of microbial communities modulating community structure and function; however, only a couple of tools are currently available for phage identification and analysis from metagenomic sequencing data. Here we employed the random forest algorithm to develop VirMiner, a web-based phage contig prediction tool especially sensitive for high-abundances phage contigs, trained and validated by paired metagenomic and phagenomic sequencing data from the human gut flora.

**Results:**

VirMiner achieved 41.06% ± 17.51% sensitivity and 81.91% ± 4.04% specificity in the prediction of phage contigs. In particular, for the high-abundance phage contigs, VirMiner outperformed other tools (VirFinder and VirSorter) with much higher sensitivity (65.23% ± 16.94%) than VirFinder (34.63% ± 17.96%) and VirSorter (18.75% ± 15.23%) at almost the same specificity. Moreover, VirMiner provides the most comprehensive phage analysis pipeline which is comprised of metagenomic raw reads processing, functional annotation, phage contig identification, and phage-host relationship prediction (CRISPR-spacer recognition) and supports two-group comparison when the input (metagenomic sequence data) includes different conditions (e.g., case and control). Application of VirMiner to an independent cohort of human gut metagenomes obtained from individuals treated with antibiotics revealed that 122 KEGG orthology and 118 Pfam groups had significantly differential abundance in the pre-treatment samples compared to samples at the end of antibiotic administration, including clustered regularly interspaced short palindromic repeats (CRISPR), multidrug resistance, and protein transport. The VirMiner webserver is available at http://sbb.hku.hk/VirMiner/.

**Conclusions:**

We developed a comprehensive tool for phage prediction and analysis for metagenomic samples. Compared to VirSorter and VirFinder—the most widely used tools—VirMiner is able to capture more high-abundance phage contigs which could play key roles in infecting bacteria and modulating microbial community dynamics.

**Trial registration:**

The European Union Clinical Trials Register, EudraCT Number: 2013-003378-28. Registered on 9 April 2014

**Electronic supplementary material:**

The online version of this article (10.1186/s40168-019-0657-y) contains supplementary material, which is available to authorized users.

## Introduction

Viruses are essential constituents of microbial communities contributing to their homeostasis and evolution. The viral community in the human gut flora is dominated by bacteriophages [1]. Phages can modulate the structure and function of a bacterial community through horizontal gene transfer (HGT) [2], thereby altering the bacterial phenotypes including virulence, antibiotic resistance, and biofilm formation [3–5]. Such phage-induced alterations could pose potential health risks by influencing bacterial pathogenicity and antibiotic resistance. For instance, phages affect virulence of facultative pathogens like *Vibrio cholera* [6]; several phage-encoded virulence factors have been discovered such as Shiga-like toxin [7], which was shown to induce apoptosis in many cell types [8, 9]. Meanwhile, the role of phages in the proliferation of antibiotic resistance is still controversial. Traditionally, phages were considered as a genetic reservoir for bacterial adaptations under stress, such as antibiotic treatment. It has been demonstrated experimentally that antibiotic-resistance genes were highly enriched in phages from antibiotic-treated mice [10]. However, in a recent study [11], researchers concluded that the presence of antibiotic resistance genes (ARG) was vastly overestimated in phage genomes. Through an exploratory bioinformatics strategy, they identified two known and 421 newly predicted ARGs in 1181 publicly available phage genomes. However, their experimental tests expressing four predicted ARGs in *Escherichia coli* did not lead to increased antibiotic resistance. The inconsistent findings indicate that the role of phages in the proliferation of antibiotic resistance and human health remains poorly understood.

Even though bacteriophages represent a significant part of health-related viral communities, to get a deeper understanding of phages remains challenging due to the difficulties in virus isolation and purification [12–14]. Yet, the use of metagenomic sequencing, which generates simultaneously genome reads from both prokaryotic cells and viruses, has promoted the viral studies dramatically by recovering phage genomes from metagenomes of ecological and clinical samples [15–17]. Using this approach, a set of potential gut-specific *Bacteroidales*-like phages was identified within human gut microbial genomes [17], which also encode antibiotic resistance genes. Another study classified and quantified the phage taxa contained within fecal metagenomes from 207 individuals worldwide using taxon-specific marker genes [18] and found differences in the abundances of particular phage taxa across human populations. Therefore, the taxonomic and functional compositions of phageome and the phage-host interactions could be revealed using directly the metagenomic data. Furthermore, a better ecological understanding of the microbiomes and deeper insights of their impacts on human health could be achieved.

Identifying phage contigs from mixed bacterial and phage sequences is a necessary and critical step for phage analysis in metagenomic studies. Most of the current tools for identifying phage sequences or prophage regions, including Phage Finder [19], Prophage Finder [20], Prophinder [21], and PHAST [22], are only suitable for virome sequencing or prokaryotic genome sequencing data, but not designed for identifying phage sequences from metagenomic data and cannot efficiently separate phage and bacterial sequences from microbiome. Metaphinder [23] is a web server developed to process metagenomic sequencing data to identity phage contigs. Nevertheless, all these aforementioned tools identify phage sequences through homology search against known phage sequences in current databases. As it is estimated that there are 10^31^ viral particles infecting microbial communities, only a few thousand viral genomes are deposited in the current databases [24, 25]. Thus, the current tools might ignore a large number of unknown or uncultured phages.

To achieve the prediction of unknown phages from metagenomic data, VirSorter [26] and VirFinder [12] were recently developed. VirSorter has employed two reference databases of viral protein sequences to detect the presence of “hallmark” genes defined as text matching “major capsid protein,” “portal,” “terminase large subunit”, “spike,” “tail,” “virion formation,” or “coat” annotations for each metagenomic contigs. Besides, VirSorter uses other metrics including viral-like genes, Pfam-affiliated genes, short genes, and depletion in strand switching to build a probabilistic model to measure the confidence level of the predicted viral region. Based on this, the metagenomic contigs can be classified into three categories: sequences having significant enrichment in viral-like genes and viral hallmark genes detected (“most confident”), sequences having either significant enrichment in viral-like genes or viral hallmark genes detected (“likely”), and sequences dissimilar with known virus references but structurally similar with known viral genomes (“possible”). In contrast, VirFinder empirically hypothesizes that viruses and phages have discernibly different k-mer frequency, so a k-mer-based machine learning model was built to determine viral signals in metagenomic samples. Both tools have presented good predictive performance. However, the performance evaluation was based on simulated metagenomes [12, 26], which were generated by artificially setting the proportion of viral contigs, thus it cannot reflect the predictive ability on actual samples. Our analysis revealed that the composition used to evaluate the aforementioned tools is significantly different from the real microbiome composition in the human gut, which may result in biased predictions. Besides, the functions offered by VirSorter and VirFinder for phage analysis within microbial communities are relatively limited; after identifying phage contigs, no further analysis is provided, such as phage-host interaction, which may reveal the key role of phages in response to particular stress. Therefore, a more powerful tool is needed to provide a deeper understanding of the possible role of phages within microbial communities.

Here, we developed VirMiner, a user-friendly web tool that employs the random forest model to identify phage contigs, especially for high-abundance phage contigs, from metagenomic data. To achieve higher predictive power in real metagenomic data, VirMiner was trained and evaluated by a human gut microbial metagenomes, with paired phageomes from purified phage libraries generated from 10 individuals treated with antibiotics and longitudinal sampling. Moreover,VirMiner provides a comprehensive analysis pipeline which includes several highlights: (1) raw reads processing, on-site metagenome assembly, and gene prediction; (2) comprehensive functional annotations including Pfam, KEGG orthology (KO), phage orthologous groups (POG), viral protein families, and viral hallmarks; (3) a highly sensitive random forest (RF) predictive model for phage contig identification, which shows outstanding performance in the identification of high-abundance phage contigs; (4) phage-host relationship prediction and CRISPR-site recognition; and (5) statistical comparisons between different sample groups.

## Materials and methods

### Updated POG database

Our updated phage orthologous groups (uPOGs) database was built in October 2016 using the same methodology as Kristensen et al. [27], except that we integrated more recently released and published phage genomes to gain more POGs. The sequences of 3319 publicly available phage genomes were acquired from the NCBI nucleotide (https://www.ncbi.nlm.nih.gov/nuccore/?term=) using same keywords as Kristensen et al. [27], besides, 759 prophage genomes were downloaded from ACLAME database [28]. These phage genomes used for uPOGs construction are available in our website (http://147.8.185.62/VirMiner/downloads/phage_genome/). The sequences of 7734 available prokaryotic genomes deposited in NCBI were also downloaded. POGs were constructed using the standard COG-building method [29].

Among these constructed POGs, we further identified virus-specific POGs based on virus quotient (VQ) computation [27]. All POGs were mapped against phage genomes and prokaryotic genomes, respectively, using psi-blast (*E* value ≤ 0.001, bit score > 40, homologous region ≥ 40 amino acids). The prokaryotic genomes with prophage regions as identified by PhiSpy [30] were excluded from the virus quotient (VQ) calculation. For each POG, VQ was calculated as the quotient of the frequency of matches to viral genomes versus the sum of the frequency of matches to both viral and prokaryotic genomes. POGs with VQ > 0.85 were considered as highly virus-specific POGs. We also identified taxa-specific POGs that can be used to detect the presence of specific taxon groups. For the POG database developed by Kristensen et al. [27] (POG 2012), results were presented using two different criteria: (a) 100% recall, 100% precision, and a VQ of 1.0 and appeared in only a single copy per genome in the POGs; (b) 100% precision threshold, the VQ threshold at 1.0, but using no recall threshold. In another study that employed the POG database for human gut microbiome analysis [18], Waller et al. used the criteria of 100% precision, VQ greater than 85%, recall greater than 85%, and being present in a single copy per genome. Here we followed the criteria of Waller et al. [18] for taxon signature POG identification. The update of POGs database (uPOGs) is available on our website (http://147.8.185.62/VirMiner/downloads/updated_POG_database/). A more detailed description of these methods is available in Kristensen et al. [27].

### Ethics statement

Written informed consent was obtained from all participants involved in the study. The study was approved by the local ethics committee in Region Zealand, Denmark (REG-026-2014) and performed in accordance with the Good Clinical Practice principles and the Helsinki declaration. Details of the study were published before study start at the clinical trials register (www.clinicaltrialsregister.eu; EudraCT nr.: 2013-003378-28).

### Study design of training dataset

A total of 10 healthy individuals were included in this study. After randomization, eight individuals were treated with different antibiotics (azithromycin for 5 days (*N* = 2), doxycycline for 7 days (*N* = 2), cefuroxime for 5 days (*N* = 2), or ciprofloxacin for 5 days (*N* = 2)), and two individuals referred to as controls received no medication (Additional file 1: Table S1 for subject information). Fecal samples were obtained at various time points: one time point before (15 days before treatment), two time points during (third and fifth day of antibiotic treatment ± 1 day), and three time points after treatment (15, 30, and 90 days after treatment ± 1 day). Control delivered fecal samples with similar intervals. In total, 59 samples were used for sequencing both microbial metagenomes and paired phageomes (for one sample collected at 15 days post cefuroxime treatment high-quality libraries could not be obtained). Samples used in this study are identical to those in Kang et al. (in preparation), therefore the metagenomic and phageome sequencing data will be released together with the companion manuscript.

### Microbial DNA extraction

DNA was extracted from 5-g aliquots of frozen stool using the MO BIO PowerMax Soil DNA Extraction Kit (MO BIO Laboratories, Inc) according to the manufacturer’s protocol with a few modifications. As a control for DNA extraction, *E. coli* MG1655 carrying pZE21 plasmid supplemented with mCherry gene was used. Overnight *E. coli* culture (37 °C, 180 rpm) in LB broth was prepared. Five milliliters of overnight culture was used for DNA extraction following the same protocol used for DNA extraction from fecal samples. DNA samples were stored at − 20 °C.

### Microbial DNA purification

After extraction, the DNA was purified (PowerClean® Pro DNA Clean-Up Kit, MO BIO Laboratories, Inc.) according to the manufacturer’s protocol. When necessary, the isolated DNA was concentrated to > 50 ng/uL using a vacuum concentrator (Concentrator plus, Eppendorf). The quantity of the DNA was measured using Qubit® 2.0 Fluorometer (Thermo Fisher Scientific Inc.). The quality of the DNA was measured using NanoDrop ND-1000 spectrophotometer (Thermo Fisher Scientific), and the size was examined by gel electrophoresis, analyzing 5 μl of the DNA on a 1% (*w*/*v*) agarose gel, containing RedSafe™ Nucleic Acid Staining Solution (iNtRON Biotechnology).

### Microbial DNA library preparation and sequencing

DNA samples were sent to Macrogen (South Korea) for library preparation and sequencing (Illumina Hiseq 2000 PE125). DNA library for sequencing was prepared by using TrueSeq Nano 550 bp kit (Illumina). Two hundred nanograms was used as input template according to supplied kit instructions. Sequencing depth was set up to a minimum of 6 Gb of data per sample.

### Phage DNA extraction

Phage particles were isolated from 5-g aliquots of frozen stool. Fifty milliliters of PBS containing calcium and magnesium was added to the aliquots, and the samples were homogenized on vortex for 20 min at highest speed (SI-H506, Horizontal 50-mL Tube Holder, Scientific Industries). Then, the samples were centrifuged three times at 4 °C: 2 min at 872×*g*, 10 min at 3800×*g*, and 20 min at 7500×*g*. After each centrifugation step, the supernatant was transferred into new 50-ml Falcon tube and the pellet was discarded. Ten milliliters of the supernatant was filtered through 0.22-μm filters (EMD Millipore Sterivex-GP SVGPL10RC Polyethersulfone Filter Unit, Millipore). To concentrate the virus particles, the filtered supernatant was concentrated to 1 ml by centrifugation in 100 Da Amicon Ultra filters (Amicon Ultra-15 Centrifugal Filter Units, Millipore) at 3488×g at 15 °C. The supernatant was filtered through a 0.45-μm syringe filter (Cellulose acetate membrane syringe filter, Filter Technology) into a 1.5-ml phase lock gel tube (5 PRIME), and 40 μL of lysozyme (10 mg/mL, Sigma-Aldrich) was added, and the filtrate was incubated for 30 min at 37 °C under shaking at 300 rpm. After incubation, 400 μL of chloroform was added to the samples and the samples were incubated for 15 min at room temperature. The samples were centrifuged at 14.000×*g* for 5 min at room temperature, and the supernatant was transferred to a 1.5-ml Eppendorf tube. A mix of DNases and RNase containing 500 U of bovine pancreas DNase I recombinant (Roche), 33 U of Baseline-ZERO™ DNase (Epicenter), 6 U of Salt Active Nuclease (ArcticZymes), and 500 U of RNase A (Roche) were added to the samples with 100 μl of 10× incubation buffer (Roche). The samples were incubated at 37 °C for 90 min, and then, at 75 °C for 10 min. After the DNase/RNase treatment, the phage particles were stored overnight at 4 °C. The phage DNA was extracted using Phage DNA Isolation Kit (46,850, Norgen Biotek) according to the manufacturer’s protocol. As a control sample for phage DNA extraction, 15 ml of lambda phage lysates sample was used. The quantity of the DNA was measured using Qubit® 2.0 Fluorometer (Thermo Fisher Scientific Inc.). Phage DNA samples were stored at − 80 °C.

### Preparation of phage lysate

A 100-ml culture of lambda phage lysogen *E. coli* K-12 strain MC1061 was grown from single colonies in LB broth at 37 °C, 180 rpm. Phage lambda was labeled with spectinomycin resistance gene *aadA* (WP_010891332.1) (Fogg et al. 2010). Upon reaching the exponential growth phase, determined by an optical density at 600 nm of 0.4, mitomycin C (Sigma) was added to a final concentration of 0.5 μg/ml. Lysogenic cultures were incubated overnight at 37 °C, 180 rpm in the dark. The cultures were centrifuged at 4.000×*g* for 10 min, and the supernatants were filtered through low protein binding 0.22-μm pore size membrane filters (Millex-GP, Millipore, Bedford, MA). Phage lysate was stored at 4 °C until use.

### Phage library preparation and sequencing

Phage DNA libraries were prepared using the KAPA HyperPlus Kit (Kapa Biosystems). All steps were conducted on ice except the two cleanups, which were performed at room temperature. The concentration was measured using Qubit® 2.0 Fluorometer (Thermo Fisher Scientific Inc.), and the size of the library was examined using Bioanalyzer (Agilent 2100 Bioanalyzer system, Agilent Technologies). The library (average size between 500 and 900 bp) were pooled and sequenced on MiSeq platform (PE300).

### User data preprocessing

**Fig. 1:**
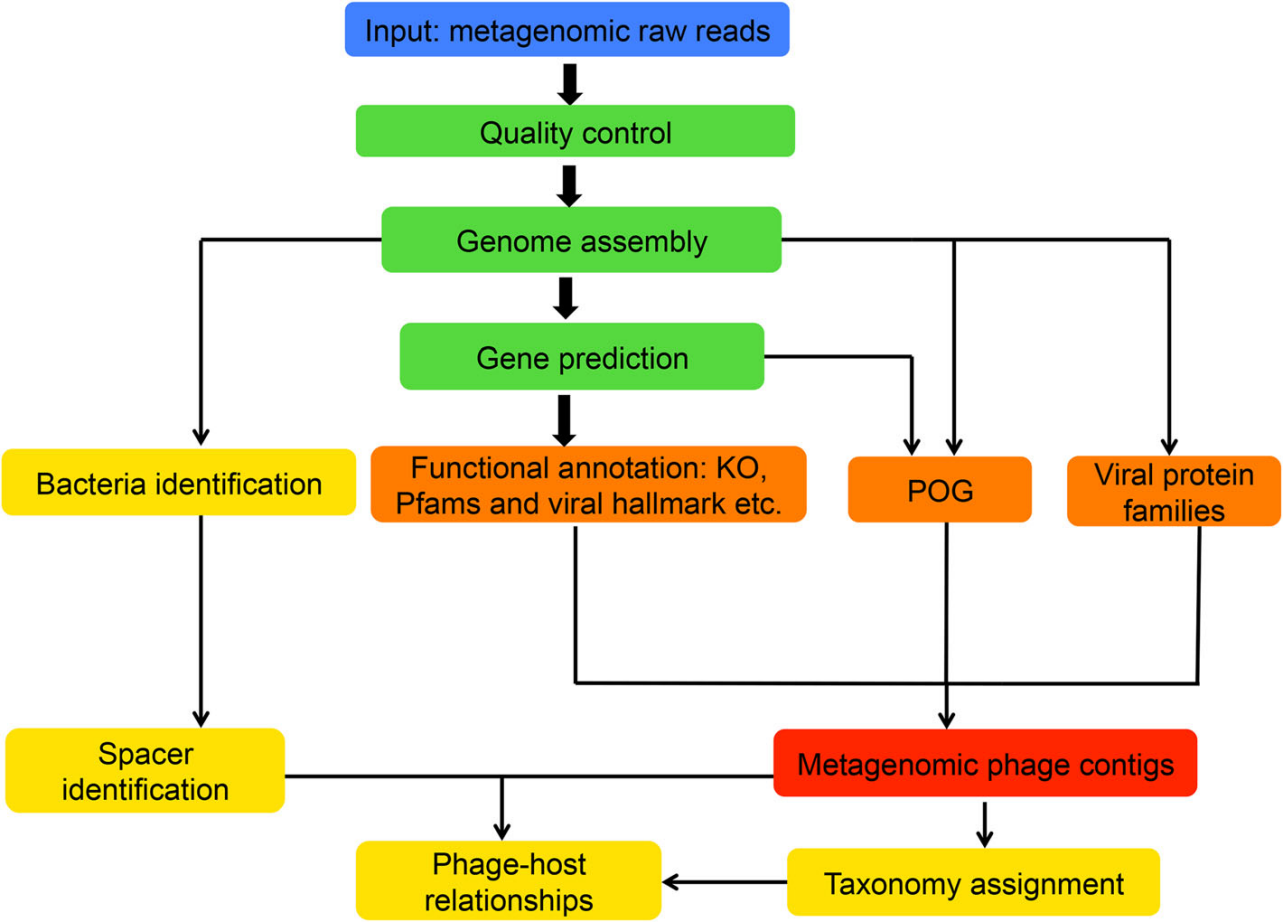
The workflow of VirMiner. The high-quality reads are assembled into contigs using IDBA_UD [33]. HMM model from GeneMark [34] was used for gene prediction. Functional profiles are generated by searching against different databases including KO, Pfam, and viral protein families defined by Paez-Espino et al. [24], POG 2012, and uPOGs. The R package randomForest was employed to identify phage contigs. The taxonomy affiliations of identified phage contigs are identified using the RDP classifier. Phage-host interaction prediction was performed using the CRISPR-spacer based method

The preprocessing of input data (metagenomic raw reads in FASTQ format) includes mainly three steps (Fig. 1): (1) for the uploaded data, adaptor region, low-quality bases/reads, and PCR-duplicated reads are removed as previously described [31, 32], the script is available on GitHub (https://github.com/TingtZHENG/VirMiner/); (2) the short reads are assembled into contigs using IDBA_UD with default parameters [33]; due to computational memory constrains of the VirMiner server, for user input data, metagenome assembly is performed independently for each sample rather than co-assembly; and (3) hidden Markov models (HMMs) from MetaGeneMark [34] are used for gene prediction.

### Functional annotation

We used different blast tools to search the predicted ORFs against multiple databases with an *e*-value cutoff of 1e-5: BLASTP against KEGG orthology (KO) [35] and viral hallmark genes [26], BLASTPGP against POG 2012 and uPOGs, and RPSBLAST against the conserved domain database (CDD) profiles [36] to annotate with Pfam [37]. The predicted genes were mapped against viral protein families [24] by hmmsearch (*E* value < 1e-5). These functional profiles were used as the metrics in building the RF model to identify phage contigs.

### Microbial metagenomes and paired phageomes used in the RF model training

The microbial metagenomes and paired phageomes were used to build the model of phage contigs identification. We defined three categories for metagenomic contigs: phage contigs, ambiguous contigs, and confident non-phage contigs. The metagenomic contigs were first mapped against the contigs from the phageomic data for the same sample using megablast (*E* value < 1e-5, identity > 98%). A metagenomic contig is marked as a phage contig if it is present in the phageomic data and meets at least one of the following criteria: (1) > 80% coverage (aligned length/the length of the query or the subject contig, which is shorter); and (2) aligned length > 10 kb. The contigs defined as ambiguous should meet one of following criteria: (1) 40–80% coverage, (2) aligned length between 4 kb and 10 kb. Other than the above two categories, the remaining ones were defined as confident non-phage contigs.

### Predictors used in the random forest (RF) model

Each contig was characterized using multiple features: (1) average depth (the number of reads mapped to a contig divided by contig length), (2) the number of predicted genes, (3) the number of genes mapped to the updated POG database, (4) the number of genes mapped to viral protein families defined by Paez-Espino et al. [24], (5) the percentage of genes annotated to viral protein families (the number of predicted genes annotated to viral protein families divided by the total number of predicted genes for this contig), (6) the number of genes mapped to KO, (7) the percentage of genes annotated to KO, (8) the number of genes mapped to Pfam, (9) the percentage of genes annotated to Pfam, and (10) the number of genes mapped to viral hallmark genes defined in Roux et al. [26]. Only contigs > 5 kb were used to build the predictive model (with the R package randomForest) and for downstream analysis.

### Predictive performance evaluation

The metagenomic datasets were split into training and test sets. A strategy similar to 10-fold cross-validation was followed to train and evaluate the predictive model. In each of the 10 steps, one of the 10 subjects was used as the test set in turn and the other nine subjects were pooled as the training set. Then the average predictive performance across all 10 subjects was computed. To overcome the imbalance of the contig classification (the putative non-phage contigs (223,021 in total) outnumbered the putative phage contigs (4515 in total)) when using RF, the training set was constructed by taking all the putative phage contigs, ambiguous contigs, and randomly selected 3000 confident non-phage contigs with abundance in the top 50th percentile. The parameters for the RF model were set as mtry = 7, ntree = 1500.

Two measurements were introduced to evaluate the predictive performance: (1) the count of contigs predicted correctly, and (2) the abundance of contigs (represented by ratio of reads mappable to contigs) that could be predicted correctly. The relative abundance of each contig was defined as the proportion of total reads mapped to the predicted contig, calculated as follows: firstly, clean reads were mapped to the predicted contig using Burrows-Wheeler Aligner (BWA) [38], then the mapped reads were identified with identity 99%, and finally the number of mapped reads per contig was divided by total reads in the sample. When evaluating the predictive performance, the putative ambiguous contigs and confident non-phage contigs were both considered as non-phage contigs.

### Phage-host interaction prediction

The CRISPR-spacer-based method is used for phage-host relationship prediction. Firstly, all the assembled contigs are mapped to prokaryotic genomes downloaded from NCBI by blastn (*E* value < 1e-5). Afterwards, the CRISPR recognition tool (CRT) [39] is employed to identify spacers from bacterial contigs. All identified spacers are searched against identified phage contigs using blastn-short function from the Blast+ package [40] (*e* value 1e^− 10^, max_target_seqs 1, identity 95%), following Paez-Espino et al. [24].

### Taxonomy affiliation for identified phage contigs

We employed the Ribosomal Database Project (RDP) classifier [41], a Naive Bayes classifier, to classify the identified phage contigs into different taxonomic levels. Originally it was developed for classifying bacterial rRNA sequences into the new bacterial taxonomy [41]. The classifier was developed based on the frequency of all possible eight-base subsequences in training set with known taxonomy information. Therefore, it is also suitable to search for the taxonomy affiliation of identified phage contigs in our case. The sequences of 3319 available phage genomes downloaded from NCBI were used to train the classifier. The taxonomy information was extracted according to the NCBI GenBank taxonomy. The identified phage contigs were assigned to different taxonomy groups using the trained model. The taxonomy assignments with estimated confidence level of ≤ 0.5 were discarded.

### Differential abundance analysis

The abundance of each phage contig is quantified using transcripts per kilobase per million reads (TPM). Wilcoxon rank-sum test is used to detect differentially abundant KO or Pfam categories when the input metagenomic samples cover two different conditions (e.g., case and control).

### Microbial diversity analysis

For the metagenomic dataset, we calculated the number of operational taxonomic units (OTUs) at both the genus and species levels for each sample based on the taxonomy affiliation of identified phage contigs assigned by RDP classifier [41]. The microbial diversity indices including Shannon index, Simpson index, and Pielou evenness index were calculated using the R package vegan [42] at both the genus and species levels. Wilcoxon rank-sum test is employed for inter-group comparisons.

### Application of VirMiner in an independent dataset

The metagenomic dataset from Raymond et al. [43] containing 24 healthy individuals was used. Eighteen individuals out of the 24 were treated for 7 days with cefprozil, and six individuals, unexposed to antibiotics, referred to as controls. Fecal samples were obtained at various time points: before treatment (exposed 0 and control 0), at the end of treatment (exposed 7 and control 7), and 90 days after the end of the treatment (exposed 90 and control 90). The metagenomic raw reads were downloaded from the European Nucleotide Archive database in the project PRJEB8094.

## Results

### Updated phage orthologous groups (uPOGs) database

The majority of the POGs in the database initially developed in 2012 by Kristensen et al. [27] (POG2012) are virus-specific, with low homology regions compared to prokaryotic genomes. Moreover, POG2012 also identified the taxon signature genes of phages. Therefore, it is suitable to be used for functional annotation of phage proteins, identification of phages, and specific phage taxon groups within mixed metagenomic data. With rapid increase in phage genomes, updating the POG resource is necessary to keep pace with the data generation. In an updated POG database (pVOGs) [44] that was published in 2016, the identification of virus-specific POGs and taxon signature genes is missing. Thus, in uPOGs, we integrated more recently released and published phage genomes to identify virus-specific POGs and taxa-specific marker genes using the previously developed method [27].

In this study, we collected 4078 phage genomes and annotated 357,460 phage proteins (“Materials and methods” section), which were subsequently clustered into 16,710 POGs. Compared to the two previous POG databases [27, 44], more orthologous groups and phage proteins were included (Table 1). We further identified virus-specific POGs that can help to distinguish prophage genes from other components in microbial genomes, based on the virus quotient (VQ), which was measured as the quotient of the frequency of matches to viral genome [27]. A POG with VQ close to 1 suggests that this POG is highly virus-specific. The distributions of VQ values in the POG 2012 and our updated POG database were comparable (Additional file 2: Table S2). In total, 11,978 virus-specific POGs were provided, which outnumbered previous databases (Additional file 2: Table S2).

**Table 1:** Statistics for different POG databases

	uPOGs	pVOGs	POG 2012
Genomes	4078	2993	1027
Proteins	357,460	295,653	97,731
POGs	16,710	9518	4542

Following the criteria of 100% precision, VQ greater than 85%, recall greater than 85%, and being present in a single copy per genome [18], we identified 640 taxon signature POGs (assigned to 32 taxon groups) which could serve as markers to identify the presence of particular taxon groups (“Materials and methods” section). Compared to previous reports (106 POGs for 40 taxa including 5 unclassified taxa) [18], more taxon marker genes were identified, while the number of taxon groups slightly decreased (32 vs 40).

### VirMiner: a comprehensive tool for phage analysis in metagenomic samples

As the workflow shown in Fig. 1, VirMiner allows users to upload metagenomic raw reads in FASTQ format, then it automatically processes the data including low-quality reads filtering, metagenomic assembly, and gene prediction. We built a predictive model to identify phage contigs based on the uPOGs and other genomic information. Firstly, co-assembly was performed by pooling all sequencing reads from different samples of the same individual for both metagenomic and phageomic data independently. A total of 3,745,889 metagenomic contigs and 41,711 phageomic contigs were yielded. We then identified 4515 putative phage contigs, 1880 ambiguous contigs and 221,141 confident non-phage contigs (“Materials and methods” section, Additional file 3: Table S3). A RF model was trained using all phage contigs, ambiguous contigs, and 3000 non-phage contigs in a manner similar to 10-fold cross-validation (“Materials and methods” section).

We used sensitivity, specificity, precision, F1 score, accuracy, and MCC (Matthews correlation coefficient) to evaluate the predictive performance of our RF model in different contig lengths (Additional file 4: Table S4, Additional file 5: Table S5, and Additional file 6: Table S6). Two measurements were used in the statistics of evaluation: the number of correctly predicted contigs and the ratio of reads mapped to the correctly predicted contigs. For contigs longer than 5 kb, our RF model showed 41.06% ± 17.51% sensitivity and 81.91% ± 4.04% specificity in the prediction of phage contigs. For the high-abundance phage contigs, the model achieved 65.23% ± 16.94% sensitivity and 79.50% ± 6.46% specificity (Additional file 4: Table S4). We also acquired similar performance for contigs longer than 1 kb and 3 kb (Additional file 5: Table S5 and Additional file 6: Table S6), indicating that VirMiner is a comprehensive tool for phage analysis from metagenomic samples.

### Comparison of VirMiner with other tools

VirMiner is a phage analysis pipeline providing various features. The functional annotation includes the prediction of KO, Pfam, viral hallmark genes, viral protein families, and POG. As described above, VirMiner uses a prebuilt predictive model to identify phage contigs from all metagenomic contigs and performs downstream analyses including phage-host relationship prediction, taxonomy analysis, and inter-group comparisons. The functionality comparison of different tools, such as VirSorter and VirFinder (Table 1), reveals that VirMiner is a powerful and user-friendly web server, which is easy to be handled especially for researchers without strong programming skills.

Furthermore, VirSorter and VirFinder were previously evaluated using simulated metagenomes that were generated by sampling from reference genomes and arbitrary setting the proportion of viral contigs. More specifically, the simulated metagenome dataset in VirSorter was generated by sampling from bacterial genomes and viral genomes available in public databases, which represent a small proportion of phage contigs, as most phage contigs in natural metagenomes have not been annotated [24, 25]. In VirFinder, each contig of simulated metagenome was definitively assigned as prokaryotic (88%), viral (10%), or ambiguously chimeric (1.8%) [18]. We further illustrated the different compositions of simulated metagenomic data used in VirFinder and our real metagenomic data generated here from eight individuals under antibiotic treatment and two controls (Additional file 3: Table S3). The average proportion of phage contigs was approximately 2% (putative phage contigs/all contigs), which was lower than in the simulated metagenomics dataset.

**Fig. 2:**
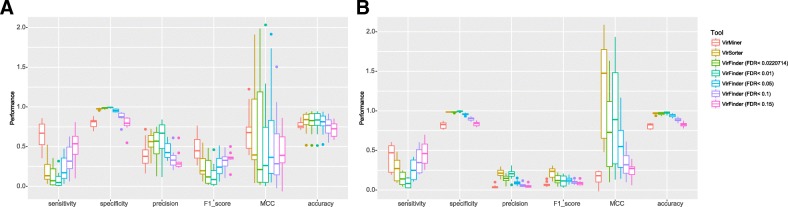
Performance comparison of VirMiner, VirSorter, and VirFinder for long contigs (> 5 kb). The predictive performance was measured based on reads ratio of correctly predicted contigs (**a**) and the number of correctly predicted contigs (**b**). The predictive performance of VirFinder was evaluated with five different cutoffs including four commonly used cutoffs (FDR < 0.01, 0.05, 0.1, and 0.15) and the value at which VirFinder can achieve the same false positive rate (FPR) as VirSorter (FDR < 0.0220714)

The predictive performances of the three tools (VirMiner, VirSorter, and VirFinder) were evaluated using our co-assembled metagenomic and phageomic contigs in different lengths (Table 2, Fig. 2, Additional file 7: Table S7, Additional file 8: Table S8, and Additional file 9: Table S9), since both VirFinder and VirSorter show different predictive abilities for contigs with different lengths [12]. As VirFinder only outputs a score indicating the possibility of the contig belonging to phage, statistical measure *p* value and corresponding corrected *p* values (FDR), users have to arbitrarily set a threshold to determine a list of identified phage contigs. We used five different cutoffs including four commonly used cutoffs (FDR < 0.01, 0.05, 0.1, and 0.15) and the value at which VirFinder can achieve the same false positive rate (FPR) as VirSorter (Table 2 and Table 3). For contigs longer than 5 kb, when we focused on the sensitivity and specificity measured based on reads ratio mapped to correctly predicted contigs, VirMiner showed higher sensitivity (65.23% ± 16.94%), precision (40.04% ± 17.46%), F1 score (0.47 ± 0.16), accuracy (77.33% ± 4.95%), and MCC (Matthews correlation coefficient) (0.70 ± 0.28) compared to VirFinder, at almost the same specificity (79.50% ± 6.46%) (Additional file 7: Table S7). In the comparison with VirSorter, VirMiner showed much higher sensitivity and F1 score but slightly lower specificity and accuracy, with lower precision and nearly the same MCC. In terms of the number of correctly predicted contigs, VirMiner showed slightly lower performance than VirFinder when FDR was set to 0.15 (Additional file 7: Table S7). VirSorter showed much higher specificity, precision, F1 score, MCC, and accuracy but much lower sensitivity. For contigs longer than 1 kb and 3 kb, similar numbers were obtained (Additional file 8: Table S8 and Additional file 9: Table S9). These results indicated that VirMiner is more sensitive to identify high-abundance phage contigs than VirSorter and VirFinder.

**Table 2:** Comparison of functionality for VirMiner (developed here), VirFinder [12], VirSorter [26], and iVirus [56]

Tools	Input raw reads	Functional annotation	Phage contig identification	Inter-group comparison	Phage-host relationship prediction	Taxonomy analysis
VirMiner	✔	✔	✔	✔	✔	✔
VirSorter	✖	✔	✔	✖	✖	✖
VirFinder	✖	✖	✔	✔	✖	✖
iVirus	✔	✔	✔	✖	✖	✔

**Fig. 3:**
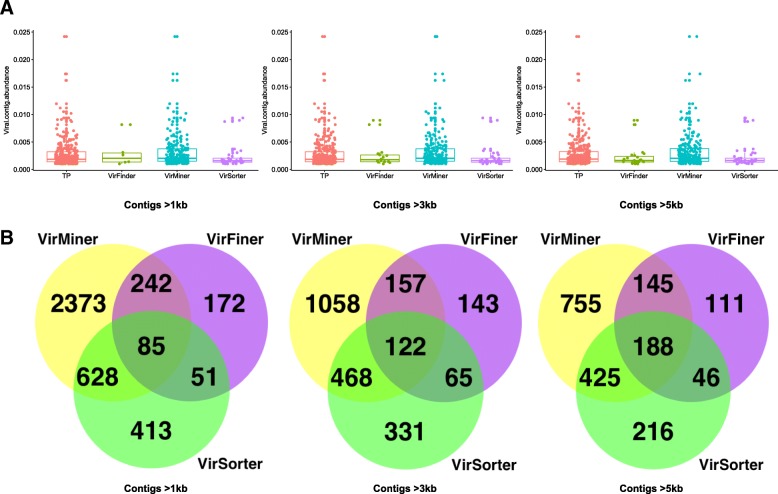
True positive phage contigs identified by VirMiner, VirSorter, and VirFinder for contigs > 1 kb, 3 kb, and 5 kb. Box plots showing abundances of true positive phage contigs identified by the three tools (**a**). Only true positive phage contigs with relative abundance > 0.001 were selected for visualization. Abundance of phage contigs referred to the ratio of reads represented by contigs over total reads in the sample. Venn diagram showing the overlap of true positive phage contigs identified by the three tools, for contigs > 1 kb, 3 kb, and 5 kb, respectively (**b**)

We also examined the prediction ability of the three tools on the identification of high-abundance phage contigs. VirMiner captured more highly-abundance phage contigs than the other two tools (Fig. 3a). In addition, we investigated the consistency of the true positive contigs identified by the three tools (Fig. 3b). For long contigs (> 5 kb), VirMiner identified 1513 true positive phage contigs, which is remarkably more than 875 and 490 contigs identified by VirSorter and VirFinder, respectively (with FDR < 0.0220714, at the same FPR as VirSorter). Notably, 67.96% (333) of phage contigs identified by VirFinder and 70.06% (613) by VirSorter were included in the list of 1513 phage contigs that VirMiner identified (Fig. 3b). The results for contigs > 1 kb and contigs > 3 kb also revealed that VirMiner detected the most true positive phage contigs among the three tools, and more than 50% of true positive phage contigs identified by VirSorter and VirFinder were captured by VirMiner. These results indicate that VirMiner has outstanding performance in the high-abundance phages.

### Application of VirMiner in an independent cohort of humans treated with antibiotics

VirMiner was applied to an independent cohort of previously published human gut microbiome samples [43]. The samples treated with antibiotics can be divided into three groups (“exposed 0,” “exposed 7,” and “exposed 90”). Correspondingly, the control samples also had three subgroups (“control 0,” “control 7,” and “control 90”). A total of 5,075,513 contigs were obtained and 458,347 long contigs (> 5 kb) were retained for downstream analyses. Among these contigs, 62,253 were identified as phage contigs (Additional file 10: Table S10). Further, 56,759 contigs were assigned to six genera by the RDP classifier including *T4virus*, *Rslunavirus*, *Phikzvirus*, *Spn3virus*, *Bxz1virus*, and *Agrican357virus*. In addition, 33,510 contigs were assigned to 11 species, with estimated confidence level of > 0.5.

**Fig. 4:**
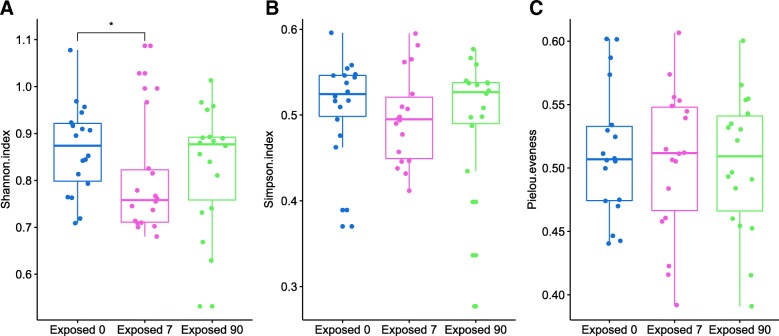
Box plot showing genus-level microbial diversity of phages based on the independent cohort of humans treated with antibiotics. The microbial diversity was measured by diversity indices including the Shannon index (**a**), Simpson index (**b**), and Pielou eveness index (**c**). Data are shown based on three conditions before treatment, at the end of treatment and at 90 days after the end of treatment. The alpha diversity of before-treatment (“exposed 0”) phage communities was significantly higher compared to group exposed to antibiotics for 7 days (“exposed 7”) (Wilcoxon test; Shannon index, *p* < 0.044)

We estimated the microbial diversity at the genus level and observed that the alpha diversity of the phage communities in baseline (“exposed 0”) was significantly higher than the samples exposed to antibiotics for 7 days (“exposed 7”) (Wilcoxon test, Shannon index: *p* < 0.044) (Fig. 4a). No significant difference of alpha diversity was observed in the other two comparisons: “exposed 0” vs. “exposed 90” and “exposed 7” vs. “exposed 90”. These results indicated that the alpha-diversity of phage communities recovered to almost the pre-treatment status at 90 days after the end of the treatment. The taxonomic eveness within phage communities was not significantly different among these three groups (Fig. 4c). No significant differences in microbial diversity were observed among different time points in non-exposed participants.

We found more functional categories that have differential abundance between time points in antibiotic-exposed participants than in control individuals. In the comparison to functional categories between “exposed 0” vs “exposed 7” samples, 122 KO groups and 118 Pfam groups were found with differentiated abundances (Wilcoxon rank-sum test, *p* < 0.05) (Additional file 11: Table S11). Among these functional groups, some are highly relevant to phages including CRISPR, multidrug resistance, and protein transport. Such alterations in these functional groups were possibly induced by antibiotic treatment [45, 46]. On the other hand, in control individuals, only 15 KO groups and 12 Pfam groups had differential abundance in the comparison between “control 0” and “control 7” (*p* < 0.05). Moreover, these functional groups were mainly associated with glutathione metabolism, carbon metabolism, alanine metabolism, and cell motility. These results indicated that VirMiner could capture the phage functions altered by exposure to different conditions such as antibiotic treatment.

**Fig. 5:**
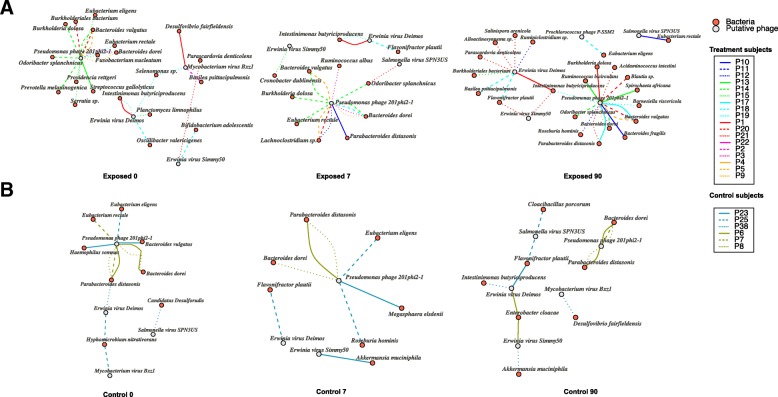
The phage-host interaction network produced from the independent cohort of humans treated with antibiotics at phage species level. The phage-host network was produced from antibiotics-exposed subjects that were divided into three groups (“exposed 0,” “exposed 7,” and “exposed 90”) (**a**) and control subjects that also had three groups (“control 0,” “control 7,” and “control 90”) (**b**). Phage (red) and bacteria (gray) are connected if the bacteria species were predicted as the host of phages. The edge color indicates the individual’s id where the phage-host interaction was observed

In the phage-host relationship prediction, 49,177 CRISPR-spacers were identified from 3,248,978 bacterial contigs and 2766 identified spacers showed exact matches with identified phage contigs. VirMiner predicted a total of 188 phage-host contig pairs from these results. Then the taxonomic classification of phage contigs and bacterial contigs were combined from the output of VirMiner to produce the phage-host interaction network (Fig. 5 and Additional file 19: Figure S1). In the antibiotic-treated participants, VirMiner identified six phage species with 35 different bacterial hosts (Additional file 12: Table S12). Except for *Prochlorococcus* phage P-SSM2 that was uniquely identified in participant P18 at the 90th day after the end of treatment, five phage species were also identified in the control samples, most of which belong to *Pseudomonas* phages and *Erwinia virus* phages. They all have a very broad host range, for example, *Pseudomonas* phages are connected with bacterial taxa from different orders including *Bacteroidales*, *Clostridiales*, *Enterobacterales*, and *Burkholderiales.* It is consistent with previous reports that *Pseudomonas* phages infect their hosts *Pseudomonas* and bacteria from other orders such as *Burkholderiales* [47], which have been suggested to be positively correlated to their metabolic diversity and multiple antibiotic resistance [48, 49]. Some of these phages have already been incorporated into phage therapy cocktails and are continuously being examined for novel therapeutic applications [50, 51].

In addition, our results also indicated that some phage-host interactions were highly specific to an individual. For example, the associations of *Pseudomonas* phage 201phi2-1 with the hosts *Bacteroidales* bacterium and *Odoribacter splanchnicus* were observed across multiple samples derived from P14. The interaction between *Pseudomonas* phage 201phi2-1 and *Ruminococcus bicirculans* was captured only in P13. Another case was the connection between *Erwinia virus Deimos* and *Alloactinosynnema* sp. from P21. These findings reveal that particular patterns of phage-host interactions, which are not affected by antibiotics treatment, are present only in certain individuals, consistent with the findings of personalized microbiomes observed at the prokaryotic and virus level in previous studies [1, 52, 53].

We also examined the dynamics of phage-host interactions in response to antibiotic treatment. At “exposed 7” sample, the connection between *Pseudomonas* phage 201phi2-1 and *Lachnoclostridium* sp. was observed in three participants (Fig. 5a). Notably, Raymond et al. also reported that *Lachnoclostridium* significantly increased in 16 of the 18 antibiotic-exposed participants [43]*.* Therefore, we inferred that *Pseudomonas* phage may be substantially impacted by antibiotic therapy. We also found some phage-host associations to remain stable throughout the time points in the control samples. For example, the association of *Pseudomonas* phage 201phi2-1 with the hosts *Parabacteroides* was observed across various time points (“control 0,” “control 7,” and “control 90”) in individuals P6 and P8 (Fig. 5b)*.*

## Discussion

Phages are believed to act as modulators of microbiota composition and agents that drive bacterial speciation in complex bacterial communities [54]. With the rapid increase of metagenomic datasets, there is a need to develop more powerful tools to analyze the taxonomic composition and functionality of phages within the microbiome communities and to achieve a deeper understanding of phage-host interactions. Currently, only a few tools are available for phage analysis in mixed phage and bacterial communities. The functionality that they provide is limited, especially the lack of in-depth downstream analysis to reveal the role of phages in metagenome and their responses to certain stress such as antibiotics treatment or disease status. We developed VirMiner to fill this gap. VirMiner identified the phage contigs, which were functionally annotated with POGs, viral protein families and viral hallmark genes, as well as commonly used databases such as KO and Pfam. Moreover, VirMiner supports two-group comparison when the input (metagenomic samples) cover different conditions (e.g., case and control). Importantly, VirMiner can predict phage-host interactions, providing novel insights into the effect of phage on pathogenicity.

VirMiner also improved the predictive ability to detect phage contigs using an RF model. VirMiner is able to capture more highly-abundance phage contigs compared to VirSorter [26] and VirFinder [12]. When evaluating the predictive performance, we mainly focused on how much “abundance of phage” (represented by the ratio of reads mappable to phage contigs) could be predicted correctly, which was, in our opinion, more important than the previous metric, namely “the number of phage contigs correctly predicted.” The rationale behind the traditional measure is treating each contig as equally important. Compared to this, adopting the strategy that weights each contig based on the number of mappable reads, which is taken as a proxy of the abundance of the corresponding sequence on the contig, we believe is more biologically meaningful. VirMiner identified highly abundant phage contigs, which could play key roles in infecting bacteria and modulating microbial community dynamics.

Sensitivity and specificity are commonly used performance measurements, which approximate the probability of positive/negative (in our case, phage contigs/non-phage contigs) labels being true. For classification problems, there are inevitable trade-offs between sensitivity and specificity. In our human gut metagenome samples that were used to build the model of phage contigs identification, the putative non-phage contigs were much more than phage contigs (Additional file 3: Table S3). Thus, we attempted to improve sensitivity at the cost of slightly decreased precision and specificity. As a result, VirSorter and VirFinder tend to predict non-phage contigs more precisely while VirMiner is more sensitive to detect phage contigs with relatively high abundance at an acceptable specificity.

We also tested other strategies to identify phage contigs. According to Paez-Espino et al. [24], a metagenomic contig identified as viral should meet at least one of the following three conditions: (1) the contig has at least five hits to viral protein families, less than 20% total genes on the contig are annotated to KO terms, less than or equal to 40% total genes are annotated with Pfams, and more than 10% genes are mapped to viral protein families; (2) the number of viral protein families on the contig is equal or higher than the number of Pfams; and (3) ≥ 60% genes identified as viral protein families. When we used this strategy to identify high-abundance phage contigs, the performance showed much lower sensitivity than VirMiner at almost the same specificity, probably because for the strategy in Paez-Espino et al. [24], less information was used to characterize phage contigs compared to VirMiner (Additional file 13: Table S13). We also attempted to add k-mer frequency profiles as metrics to train the RF model. This approach increased the specificity for identification of both high-abundance phage contigs and the count of phage contigs, but the sensitivity dropped dramatically. Based on these benchmarking activities, we consider VirMiner an important tool for analyzing phage dynamics in metagenomic datasets.

We also assessed the robustness of the VirMiner predictive model when used for the identification of viral contigs from other environmental data. A subset of publicly available paired viral-microbial metagenomic data from the Tara Oceans samples (40 samples in total), which were included in the study of Sunagawa et al. [55], was used as an independent test set (Additional file 14: Table S14). The phage contigs in each metagenomic sample were identified using the VirMiner predictive model. We used the same methodology described in the manuscript to define three categories for the metagenomic contigs (phage contigs, ambiguous contigs, and confident non-phage contigs) for predictive performance evaluation. As a result, VirMiner achieved 45.85% ± 15.26% sensitivity and 92.22% ± 4.24% specificity in the prediction of phage contigs from the Tara dataset. In particular, for the high-abundance phage contigs, VirMiner reached 53.37% ± 21.91% sensitivity at high specificity (89.21% ± 8.77%) (Additional file 15: Table S15). These results indicate that the VirMiner predictive model is robust and sensitive when it is applied for the identification of viral contigs from other than human gut environmental microbiomes.

Besides the above analysis, we attempted to construct the RF predictive model using mixed Tara Ocean samples and human gut metagenomic data. The result of repeated fivefold cross-validation 10 times showed 57.05% ± 4.55% sensitivity and 80.19 ± 2.09% specificity in the prediction of phage contigs. In particular, for the high-abundance phage contigs, it showed 60.94% ± 7.31% sensitivity and 82.89% ± 2.69% specificity (Additional file 16: Table S16). Our findings indicated that the RF-based predictive model for viral contig identification is basically independent of the environmental microbiome. Another attempt was to train the RF predictive model only with the Tara Oceans metagenomic samples and test its performance with the dataset from the human gut samples. This RF model showed much lower sensitivity and specificity than VirMiner (Additional file 17: Table S17). One of the possible reasons is that there is much more noise presented in the Tara Oceans samples. Using the VirMiner pipeline, the quality control report of the metagenomic raw reads showed that 12 out of 40 samples have more than 10% low-quality sequences while in our human gut metagenomic samples that were used for the VirMiner training dataset, of 59 samples, only one sample contains more than 10% low-quality sequences (Additional file 18: Table S18 and Additional file 19). Therefore, we concluded that the predictive model of VirMiner outperformed RF-based models based solely on the Tara Oceans data.

## Conclusions

In summary, we developed VirMiner based on actual phageomics and microbial metagenomics samples to fill this gap. Compared to VirSorter and VirFinder (the most widely used tools), VirMiner is able to capture more highly abundant phage contigs, which could play key roles in infecting bacteria and influencing microbial community dynamics. Moreover, VirMiner is a comprehensive tool for phage analysis in metagenomic samples. It supports statistical comparison among different groups (e.g., case and control). Importantly, VirMiner can predict phage-host interactions, providing novel insights into the effect of phage on pathogenicity. Our example application of VirMiner to an independent cohort of previously published human gut microbiome samples highlights its utility in exploring the dynamics of functional profiles in phage communities and phage-host interactions in response to antibiotic treatment.

## Additional files

Additional file 1: **Table S1.** Sample information of human gut microbial metagenomic data and matched phageomes used in the RF training and evaluation dataset. (XLSX 43 KB) (XLSX 42 kb)

Additional file 2: **Table S2.** Virus-specific POGs in uPOGs and POG 2012. (XLSX 39 KB) (XLSX 37 kb)

Additional file 3: **Table S3.** The number of putative metagenomic phage contigs and non-phage contigs in metagenomic assembled contigs. (XLSX 41 KB) (XLSX 39 kb)

Additional file 4: **Table S4.** Predictive performance of VirMiner for contigs > 5 kb based on reads ratio of correctly predicted contigs and the number of correctly predicted contigs. (XLSX 51 KB) (XLSX 50 kb)

Additional file 5: **Table S5.** Predictive performance of VirMiner for contigs > 3 kb based on reads ratio of correctly predicted contigs and the number of correctly predicted contigs. (XLSX 50 KB) (XLSX 48 kb)

Additional file 6: **Table S6.** Predictive performance of VirMiner for contigs > 1 kb based on reads ratio of correctly predicted contigs and the number of correctly predicted contigs. (XLSX 50 KB) (XLSX 48 kb)

Additional file 7: **Table S7.** Comparison with VirMiner, VirSorter, and VirFinder for contigs > 5 kb based on reads ratio of correctly predicted contigs and the number of correctly predicted contigs. (XLSX 47 KB) (XLSX 48 kb)

Additional file 8: **Table S8.** Comparison with VirMiner, VirSorter, and VirFinder for contigs > 3 kb based on reads ratio of correctly predicted contigs and the number of correctly predicted contigs. (XLSX 53 KB) (XLSX 49 kb)

Additional file 9: **Table S9.** Comparison with VirMiner, VirSorter, and VirFinder for contigs > 1 kb based on reads ratio of correctly predicted contigs and the number of correctly predicted contigs. (XLSX 55 KB) (XLSX 50 kb)

Additional file 10: **Table S10.** The number of identified phage contigs for each sample in the independent cohort of humans treated with antibiotics. (XLSX 42 KB) (XLSX 41 kb)

Additional file 11: **Table S11.** The list of 122 KO groups and 118 Pfam groups found significantly different (Wilcoxon rank-sum test, *p* < 0.05) exposed 0 vs exposed 7 using VirMiner. (XLSX 69 KB) (XLSX 66 kb)

Additional file 12: **Table S12.** The predicted phage-host interactions in the independent cohort of humans treated with antibiotics at species level. (XLSX 52 KB) (XLSX 40 kb)

Additional file 13: **Table S13.** The predictive performance of the method for phage contig identification in Paez-Espino et al [24]. (XLSX 47 KB) (XLSX 46 kb)

Additional file 14: **Table S14.** The paired viral-microbial metagenomic data from Tara Oceans samples used in this study. (XLSX 47 KB) (XLSX 40 kb)

Additional file 15: **Table S15.** The predictive performance of VirMiner in the metagenomic data from Tara Oceans samples. (XLSX 69 KB) (XLSX 57 kb)

Additional file 16: **Table S16.** The predictive performance of the RF model trained with mixed Tara Oceans metagenomic data and human gut samples. (XLSX 46 KB) (XLSX 47 kb)

Additional file 17: **Table S17.** The predictive performance of the RF model trained only with Tara Oceans metagenomic samples in the human gut samples. (XLSX 48 KB) (XLSX 47 kb)

Additional file 18: **Table S18.** The summary of quality control statistics in the Tara Oceans metagenomic samples and human gut samples that were used for the VirMiner training dataset. (XLSX 48 KB) (XLSX 50 kb)

Additional file 19: **Figure S1.** The phage genera -host interaction network produced from the independent cohort of humans treated with antibiotics at phage genus level. (PDF 516 kb)
