# Supplementary figures and images for: Mining, analyzing, and integrating viral signals from metagenomic data
Source: Microbiome. 2019 Mar 19;7:42. doi: 10.1186/s40168-019-0657-y (PMC6425642; doi:10.1186/s40168-019-0657-y)

A

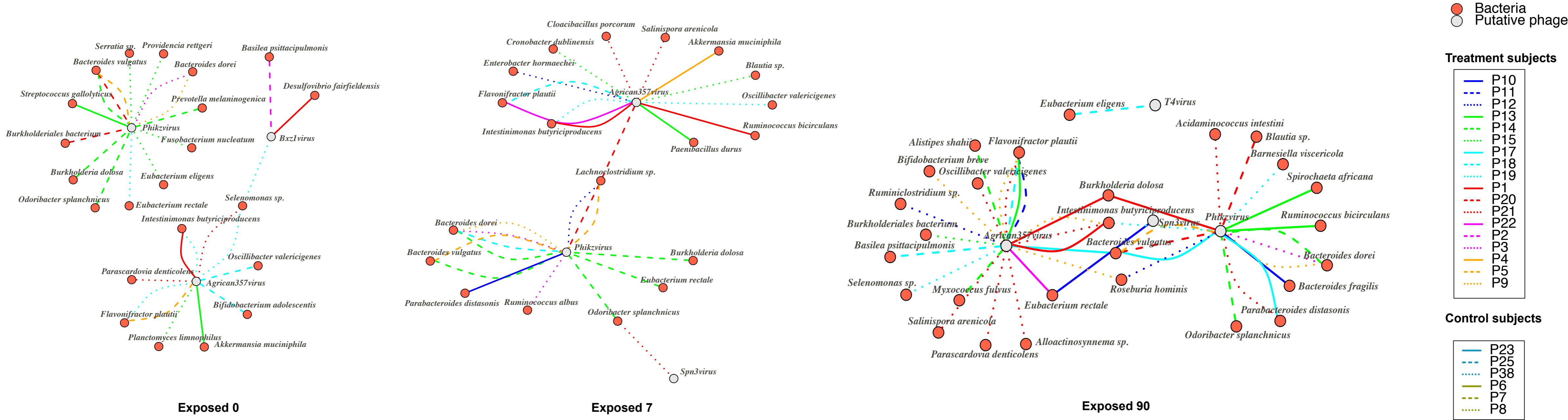

B

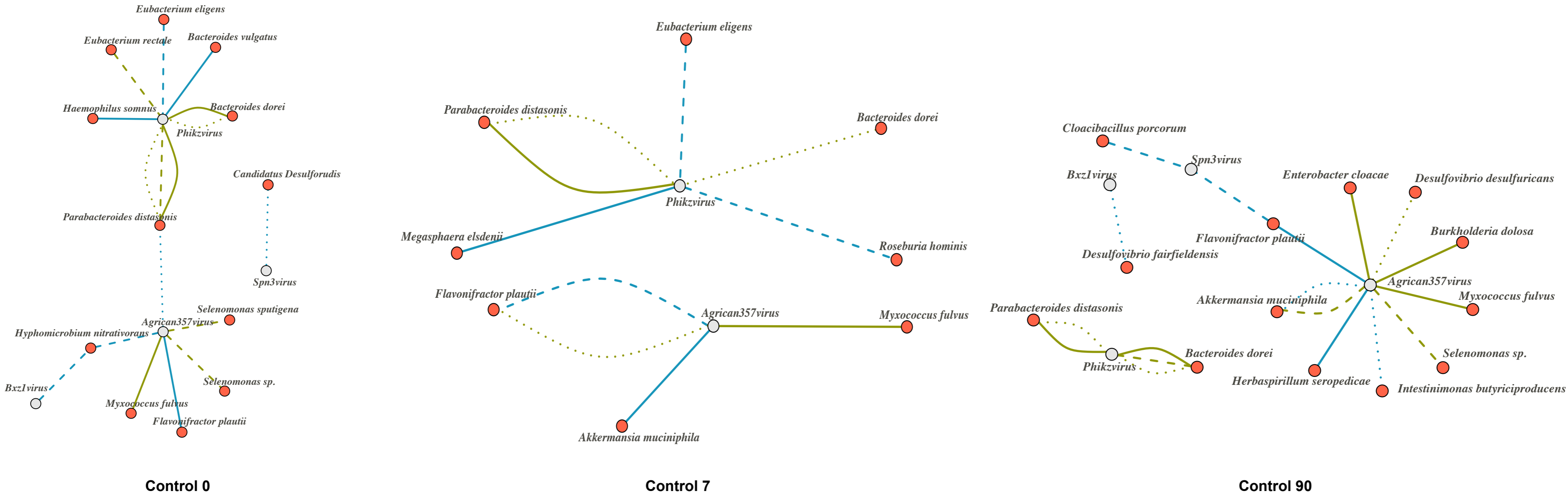

Supplement: Supplementary file 19 — Figure S1. The phage genera -host interaction network produced from the independent cohort of humans treated with antibiotics at phage genus level. (PDF 516 kb) [file 40168_2019_657_MOESM19_ESM.pdf]
